# Incidence of Vitamin D Deficiency in Foot and Ankle Arthrodesis Nonunions

**DOI:** 10.7759/cureus.57028

**Published:** 2024-03-27

**Authors:** Victor Anciano, Sterling K Tran, James B Carr, Campbell Edwards, Dylan Russell, Risa T Reid, Joseph Park

**Affiliations:** 1 Orthopaedics, University of Louisville Hospital, Louisville, USA; 2 Orthopaedic Surgery, University of Alabama at Birmingham, Birmingham, USA; 3 Orthopaedic Surgery, Hospital for Special Surgery Florida, West Palm Beach, USA; 4 Orthopaedic Surgery, University of Virginia, Charlottesville, USA

**Keywords:** vitamin d deficiency, vitamin d supplementation, ankle and foot, nonunion, arthrodesis

## Abstract

Background

Vitamin D has been found to be crucial in musculoskeletal health. The role of vitamin D levels in orthopedic patients has become a growing area of interest given its negative impact on fracture healing which can contribute to the development of nonunion following surgery. We sought to investigate the incidence of hypovitaminosis D in a cohort of patients who experienced a nonunion following a foot and ankle arthrodesis procedure.

Methodology

Patients who underwent a major elective foot and ankle arthrodesis procedure and developed a nonunion were given the opportunity to obtain serum vitamin D levels. All vitamin D levels were reported from percutaneous venous blood samples and compared to our institution’s range of accepted normal values (25-80 ng/mL).

Results

A total of 13 patients who developed a nonunion agreed to have a vitamin D level obtained, and 11 of 13 patients had a low vitamin D level (average = 14.6 ng/mL, range = 9-24 ng/mL). Five patients underwent revision arthrodesis after normalization of vitamin D levels, and four out of five patients went on to successful union.

Conclusions

Hypovitaminosis D may be a modifiable risk factor for nonunion following a major foot and ankle arthrodesis procedure. Orthopedic surgeons should consider vitamin D screening and supplementation in patients undergoing elective arthrodesis procedures.

## Introduction

Vitamin D plays an important role in bone metabolism through the actions of both calcium and phosphorus. It is a fat-soluble steroid that can be obtained via diet or synthesized within the epidermis in the presence of ultraviolet radiation [1,2]. In its active form, the main action of vitamin D is to increase gut absorption of calcium and phosphorus, which helps promote bone remodeling. Without adequate calcium and phosphorus uptake, the mineralization of the bone matrix is compromised, which can lead to the development of rickets in children and osteomalacia in adults [3].

While overt vitamin D deficiency (hypovitaminosis D) is relatively rare, subclinical vitamin D deficiency has been reported at alarming rates [4,5]. The prevalence of hypovitaminosis D in the general population has been estimated between 30% and 40% with higher rates in the African American and Hispanic populations [6-8]. Hypovitaminosis D has been associated with multiple adverse health consequences, including an increased risk of osteoporosis, falls, and fractures [9]. Recently, hypovitaminosis D has also been associated with poor clinical outcomes following orthopedic interventions, including nonunion after fracture management and fibrous union after posterior spinal fusion [10,11]. However, the relationship between hypovitaminosis D and clinical outcomes following other arthrodesis procedures, including those performed in the foot and ankle, has not been fully investigated.

Open foot and ankle arthrodesis procedures are commonly performed with increasing popularity. Between 1994 and 2006, the population-adjusted rates of foot and ankle arthrodesis procedures performed in the United States increased by 146% [12]. These surgeries are usually quite successful with reported union rates over 90% [13,14]. However, a nonunion result continues to be the leading cause of postoperative failure with rates reported between 5% and 41% [15-18]. Factors associated with nonunion include fracture type, evidence of avascular necrosis, infection, major medical comorbidities, and open injuries [16]. Additional risk factors include history of tobacco abuse, alcohol abuse, illegal drug use, psychiatric disorders, and diabetes [15].

While hypovitaminosis D has been implicated in nonunion following fracture management and posterior spinal fusion surgery, it has not been evaluated in the setting of a nonunion following a foot and ankle arthrodesis procedure. The current paper is a short retrospective case series of 13 patients who presented with nonunion after a major foot and ankle arthrodesis procedure. The objective of this paper was to evaluate the incidence of hypovitaminosis D in patients who experienced nonunion following a foot and ankle arthrodesis procedure.

## Materials and methods

A total of 32 patients who presented to our institution’s foot and ankle orthopedic surgery clinic for post-surgical follow-up after a major foot and ankle arthrodesis procedure between January 1, 2015, and December 31, 2015, were evaluated for the presence of a nonunion. A major arthrodesis procedure was defined as an ankle (tibiotalar) arthrodesis, tibiotalocalcaneal (TTC) arthrodesis, subtalar arthrodesis, “triple” arthrodesis (subtalar joint, calcaneocuboid joint, and talonavicular joint), tarsometatarsal (TMT) arthrodesis, or any combination of the aforementioned procedures. Other criteria were major arthrodesis as defined above, post-traumatic arthritis, and inflammatory arthritis. Exclusion criteria included infectious arthritis, prior attempts at arthrodesis, and acute open fractures. The study was IRB-exempt per our Institution Review Board due to the retrospective nature and no use of patient-identifying information. The study was entirely conducted at a tertiary referral center for orthopedic pathologies.

All arthrodeses were performed electively by a single foot and ankle fellowship-trained surgeon. None of the surgeries were performed in the acute trauma setting. Nonunion was defined by a lack of evidence of bony union on radiographs or computed tomography (CT) scans at least six months after surgery. Nonunion was also defined by the presence of hardware failure at any period of post-surgical follow-up with associated findings of radiographic nonunion. All radiographs and CT scans were evaluated by a board-certified, fellowship-trained musculoskeletal radiologist at our institution. Patients were excluded if they had (1) undergone an arthrodesis procedure due to trauma and (2) developed a septic nonunion.

When a nonunion was present, the patient’s vitamin D level was obtained from a percutaneous venous blood sample. Vitamin D levels were compared to the relative normal values set by our institution’s laboratory (25-80 ng/mL). Patients were also analyzed for the presence of smoking, diabetes mellitus (DM), cardiovascular disease (CAD), peripheral vascular disease (PVD), and body mass index (BMI). For the results analysis, all continuous data were expressed as mean values.

## Results

A total of 13 patients met the inclusion criteria and agreed to have vitamin D levels drawn. Four (31%) patients were male, and nine (69%) patients were female. The patients had an average age of 51 years (range = 35-76 years) and an average BMI of 37.7 kg/m^2^ (range = 24.4-49.4 kg/m^2^). Five patients were nonsmokers, two patients were current smokers, and six patients were former smokers at the time of surgery. Two patients had DM and no patients had a history of CAD or PVD (Table 1). The arthrodesis procedures performed included three tibiotalar arthrodeses, three triple arthrodeses, four TTC arthrodeses, two TMT arthrodeses, and one subtalar plus talonavicular arthrodesis (Table 2).

**Table 1:** Demographics of the patient population. F = female; M = male; Hx = history; BMI = body mass index; DM = diabetes mellitus; CAD = coronary artery disease; PVD = peripheral vascular disease

Patient number	Gender (F/M)	Age	BMI	Smoking Hx (Yes/No/Former)	DM (Yes/No)	CAD/PVD (Yes/No)
1	M	76	24.4	No	No	No
2	F	57	39.5	Former	Yes	No
3	F	35	49.4	Former	No	No
4	F	49	25.5	No	No	Yes
5	M	50	45.2	No	No	No
6	F	41	31.9	Former	No	No
7	F	35	37.1	No	No	No
8	F	55	43	Yes	No	No
9	F	56	45.8	Former	Yes	No
10	F	40	46.8	No	No	No
11	M	67	31.1	Former	No	No
12	F	40	43.9	Yes	No	No
13	M	62	26.1	Former	No	No

**Table 2:** Vitamin D levels, associated arthrodesis procedures, follow-up duration, and the presence of nonunion for each patient. TTC = tibiotalocalcaneal; TMT = tarsometatarsal; TN = talonavicular

Patient number	Surgery	Follow-up (months)	Union vs. nonunion	Broken hardware	Vitamin D level (ng/mL)	Revision surgery
1	Ankle arthrodesis	22.3	Nonunion	No	15	No
2	Left TTC fusion	29.2	Nonunion	Yes	12	Yes
3	Ankle arthrodesis	35.0	Nonunion	No	14	No
4	Triple arthrodesis	22.5	Nonunion	Yes	21	Yes
5	TTC fusion	44.6	Nonunion	No	10	No
6	TMT arthrodeses	26.7	Nonunion	No	24	No
7	Triple arthrodesis	27.3	Nonunion	Yes	14	Yes
8	Subtalar arthrodesis	31.7	Nonunion of the TN joint only	No	9	Yes
9	TTC arthrodesis	10.9	Nonunion	No	16	No
10	TMT arthrodeses	24.2	Nonunion	No	16	No
11	Triple arthrodesis	22.3	Nonunion	Yes	28	No
12	Ankle arthrodesis	29.2	Nonunion	Yes	10	Yes
13	TTC fusion	35.0	Nonunion	Yes	34	Yes

**Figure 1:**
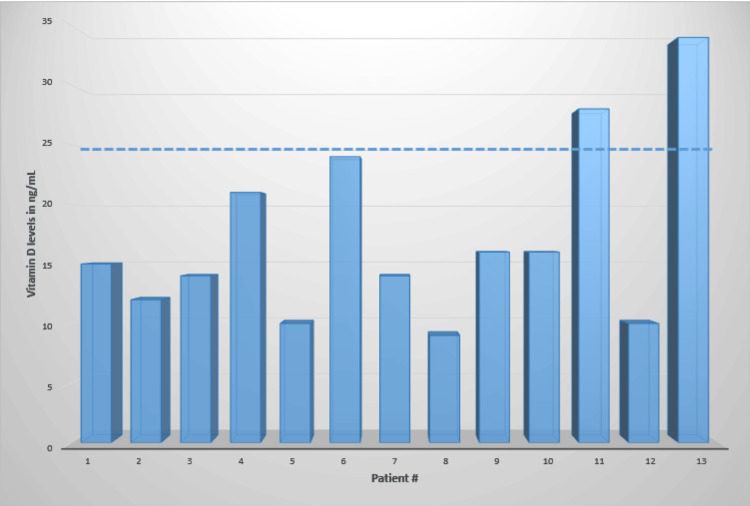
Vitamin D levels in patients with nonunions of foot and ankle arthrodeses. The dotted line represents the cutoff for normal values (25 ng/mL).

**Figure 2:**
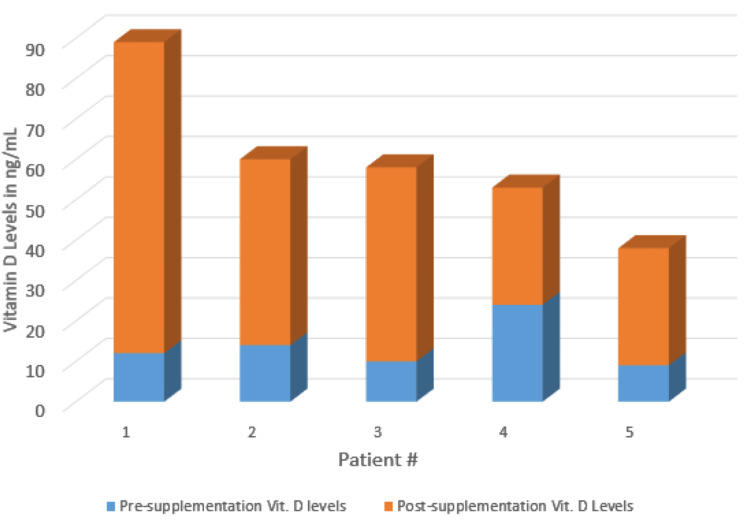
Repeat levels of vitamin D in patients who underwent vitamin D supplementation. Blue = Pre-supplementation vitamin D levels. Orange = Post-supplementation vitamin D levels.

The average vitamin D level in the 13 patients was 17.1 ng/mL (range = 9-34 ng/mL). Two patients had vitamin D levels in the lower range of normal (28 ng/mL and 34 ng/mL), while the other 11 patients had vitamin D levels below the range of normal. The average vitamin D level for the 11 patients below the accepted normal value was 14.6 ng/mL (range = 9-24 ng/mL) (Figure 1). The average length of clinical follow-up was 21.7 months (range = 10.8-43.9 months). During the follow-up period, seven patients had broken hardware on follow-up radiographs, and five patients elected to undergo revision arthrodesis (Table 1). The average time to revision surgery from the initial surgery was 10.9 months (range = 4.5-14.8 months). Five patients underwent revision arthrodesis after normalization of vitamin D levels, and four out of five patients went on to successful union (Figure 2). One patient had a failed revision arthrodesis, which ultimately resulted in the patient electing for a below-knee amputation (Table 2).

## Discussion

The exact role of hypovitaminosis D remains a subject of controversy in the setting of various orthopedic conditions, including the postoperative state. The incidence of hypovitaminosis D in various orthopedic surgery patient cohorts has been reported between 43% and 80% [19-24]. Some studies suggest that hypovitaminosis D does not significantly affect bone healing or clinical outcomes [25]. For example, Bodendorer et al. demonstrated that trauma patients with hypovitaminosis D did not have a significantly different reoperation rate compared to patients with normal vitamin D levels after undergoing orthopedic surgical intervention for acute fractures [25]. However, other studies have shown hypovitaminosis D to be correlated with inferior clinical outcomes after undergoing surgical fixation of various fracture types, including hip fractures, ankle fractures, and distal radius fractures [21,22,26]. Warner et al. recently showed that patients with preoperative vitamin D deficiency had a poorer range of motion and quality of life scores, as measured by foot and ankle outcome scores at one-year follow-up after surgical fixation of ankle fractures [26]. It is unclear if these lower functional scores were from inferior healing or other factors.

In the current case series, 84.6% of patients with a nonunion were found to have vitamin D levels below an accepted normal range. This is on the upper end of previously reported incidences within orthopedic surgery patients and is higher than any known incidence for a foot and ankle patient population. Our study mirrors a similar study performed by Michelson and Charlson, who screened vitamin D levels in patients before undergoing elective major foot and ankle arthrodesis procedures [27]. Of note, the study was performed in Vermont, where its northern location is a known risk factor for hypovitaminosis D. They found that 54 of 81 patients (67%) had hypovitaminosis D with only a 56% success rate for correcting the low vitamin D level after supplementation. Unlike this study, the clinical outcome and presence of nonunion following the procedures were not analyzed in the study.

Preoperative vitamin D levels have been an area of interest in other areas of orthopedic surgery that rely on arthrodesis procedures, including spine surgery. Kerezoudis et al. performed a systematic and critical review of the association between vitamin D deficiency and outcomes in spinal fusion surgery [28]. It was noted that patients with preoperative vitamin D deficiency achieved lower fusion rates along with a higher rate of low back pain postoperatively. Additionally, Ravindra et al. showed a significantly longer time to fusion in vitamin D-deficient groups [10]. They also discovered that hypovitaminosis D was an independent predictor of nonunion when adjusting for other risk factors. The aforementioned studies continue to support the potential role of screening for hypovitaminosis D in patients undergoing an arthrodesis procedure and treating low levels when present.

It is important to consider that risk factors for nonunion after arthrodesis procedures are numerous and not just limited to hypovitaminosis D. The most commonly cited risk factors include diabetes, smoking status, alcohol abuse, and obesity [15-18]. Rabinovich et al. analyzed these risk factors and discovered a higher risk of nonunion in patients who smoke tobacco, abuse alcohol, and have major medical comorbidities, including atherosclerosis, immunosuppression, DM, rheumatoid arthritis, and connective tissue disease [17]. Overall, smokers, patients with increased BMI, and diabetic patients have associated 80%, 70%, and 65% respective rates of major complications [17]. Other external factors such as worker’s compensation and noncompliance with postoperative instructions may contribute to a nonunion following foot and ankle arthrodesis procedures. In addition to nonunion, other adverse events associated with these risk factors include infection, bone loss, and malunion deformity.

This study contributes to the growing body of literature that advocates for the importance of vitamin D levels in patients undergoing orthopedic surgeries, including arthrodesis. Hypovitaminosis D is a controllable risk factor that can be normalized before an elective procedure, which may help reduce nonunion risk. However, it should also be noted that replenishing body reserves of vitamin D is not without difficulties. Robertson et al. have shown that despite close monitoring, success rates in normalizing vitamin D levels can be as low as 54.5% in vitamin D-insufficient groups [29]. Additionally, orthopedic surgeons have been found to be uncomfortable and less willing to monitor and replenish vitamin D levels [30]. Nonetheless, orthopedic surgeons would be prudent to normalize this controllable risk factor whenever possible before and after an orthopedic procedure that involves significant bone remodeling or high turnover of bone.

This study has multiple strengths and weaknesses. The strength of the study is its contribution to a small, yet growing, amount of literature analyzing hypovitaminosis D in patients with adverse outcomes following various orthopedic procedures. It also provides a logical next step in further elucidating the impact of hypovitaminosis D following major foot and ankle arthrodesis procedures. The first limitation is the small sample size and lack of a control group which lead to an underpowered study. As a result, we could not perform statistical analysis on the effect of additional risk factors on nonunion rates independent of vitamin D levels. Therefore, while the presence of other risk factors was relatively low within our cohort, it is not possible to associate the direct causality of failed arthrodesis with hypovitaminosis D. In addition, because vitamin D levels were only obtained for patients with a nonunion, we were unable to determine the average vitamin D level for a successful arthrodesis cohort. However, it should be noted that four out of the five patients who underwent revision arthrodesis after vitamin D supplementation had a successful fusion. Overall, the literature is scant with data analyzing surgical outcomes between patients treated versus patients untreated for hypovitaminosis D, and such a study would be a logical next step to build on current studies.

## Conclusions

Hypovitaminosis D appears to be a prevalent condition in a variety of orthopedic surgical patients, including those undergoing an elective foot and ankle arthrodesis procedure. Orthopedic surgeons should consider vitamin D levels when managing patients who are in a high bone turnover state. Studies analyzing the effect of vitamin D supplementation in patients with hypovitaminosis D who undergo a major arthrodesis procedure would be a useful contribution to the growing body of literature investigating hypovitaminosis D in orthopedic patients.

As a result of the findings from our study, we have adopted routine vitamin D testing in the preoperative evaluation for complex arthrodesis procedures. Surgery is delayed until vitamin D levels are corrected to normal levels, and oral vitamin D3 therapy is continued throughout the postoperative phase for all arthrodesis procedures.
